# Optical coherence microscopy as a novel, non-invasive method for the 4D live imaging of early mammalian embryos

**DOI:** 10.1038/s41598-017-04220-8

**Published:** 2017-06-23

**Authors:** Karol Karnowski, Anna Ajduk, Bartosz Wieloch, Szymon Tamborski, Krzysztof Krawiec, Maciej Wojtkowski, Maciej Szkulmowski

**Affiliations:** 1 Institute of Physics, Faculty of Physics, Astronomy and Informatics, Nicolaus Copernicus University, Grudziadzka 5, 87-100 Torun, Poland; 2 Department of Embryology, Faculty of Biology, University of Warsaw, Miecznikowa 1, 02-096 Warsaw, Poland; 3 Institute of Computing Science, Poznan University of Technology, Piotrowo 2, 60-965 Poznan, Poland; 4 Institute of Physical Chemistry, Polish Academy of Sciences, Kasprzaka 44/52, 01-224 Warsaw, Poland

## Abstract

Imaging of living cells based on traditional fluorescence and confocal laser scanning microscopy has delivered an enormous amount of information critical for understanding biological processes in single cells. However, the requirement for a high numerical aperture and fluorescent markers still limits researchers’ ability to visualize the cellular architecture without causing short- and long-term photodamage. Optical coherence microscopy (OCM) is a promising alternative that circumvents the technical limitations of fluorescence imaging techniques and provides unique access to fundamental aspects of early embryonic development, without the requirement for sample pre-processing or labeling. In the present paper, we utilized the internal motion of cytoplasm, as well as custom scanning and signal processing protocols, to effectively reduce the speckle noise typical for standard OCM and enable high-resolution intracellular time-lapse imaging. To test our imaging system we used mouse and pig oocytes and embryos and visualized them through fertilization and the first embryonic division, as well as at selected stages of oogenesis and preimplantation development. Because all morphological and morphokinetic properties recorded by OCM are believed to be biomarkers of oocyte/embryo quality, OCM may represent a new chapter in imaging-based preimplantation embryo diagnostics.

## Introduction

In recent years, unprecedented advancements in fluorescence live imaging have been made. These advancements, coupled with the development of molecular techniques that provide genetically modified embryos expressing fluorescently tagged proteins, have been instrumental in discovering the molecular mechanisms governing early mammalian development. Our knowledge of oogenesis, fertilization and the first embryonic divisions is incomparably greater and deeper than it was even a decade ago, allowing us to pinpoint the properties of oocytes and embryos that are crucial to ensure their high developmental potential and serve as biomarkers of oocyte/embryo quality^1^. For example, the mechanisms by which the chromatin configuration in immature oocytes or the architecture of a metaphase spindle in ovulated oocytes affect embryonic development have been thoroughly investigated^2, 3^. Similarly, as shown in many reports, the structures of nuclei (known as pronuclei) and cytoplasm in zygotes and the spatiotemporal dynamics of the first embryonic divisions can be used to predict quality of the developing embryos^1, 4^. However, until now, we typically were not able to visualize many of those properties non-invasively without fluorescent dyes or tags and sample pre-processing, thus, their usability in oocyte/embryo selection procedures at *in vitro* fertilization (IVF) clinics or animal breeding facilities has been limited.

Optical coherence microcopy (OCM) is a microscopic incarnation of optical coherence tomography (OCT) and may be used as an alternative imaging approach to live fluorescence microscopy, as it provides 3D reconstructions based on intrinsic contrasting of back-scattered coherent light^5, 6^. Unlike confocal microscopy, which requires fluorescent markers and high numerical aperture and may cause short- and long-term photodamage^7^, OCM is non-invasive and widely applicable in medicine as the primary method for 3D structural and functional imaging *in vivo* with micrometer resolution and a sampling frequency of up to tens of megahertz^8^. Previous attempts to visualize intracellular structures by OCM imaging involved the full-field OCM technique^9–12^, where in order to reconstruct 3D image of a cell, mechanical scanning along the z-axis is required and several 2D interference images need to be acquired at each depth step. Although those studies revealed some contrast between pronuclei and cytoplasm, they failed, due to the speckle noise, to show fine intracellular structures inside those compartments and did not allow for a high quality 3D cellular reconstruction. Moreover, until now the OCM has not been used to follow cellular processes in time. Here, we propose to combine scanning beam OCM that offers higher sensitivity and imaging speeds than full field OCM with custom scanning and signal processing protocols. This approach allows us to exploit the internal motion of cytoplasm to effectively reduce the speckle noise and enable high-resolution intracellular time-lapse imaging. Our OCM system provides 3D label-free images of critical intracellular organelles (nuclei with nucleoli, metaphase spindles, networks of ER and mitochondria) and, most importantly, may be used to monitor and quantitatively analyze their dynamic behavior and evolution over time.

## Results and Discussion

### Construction of the OCM system and data processing

**Figure 1:**
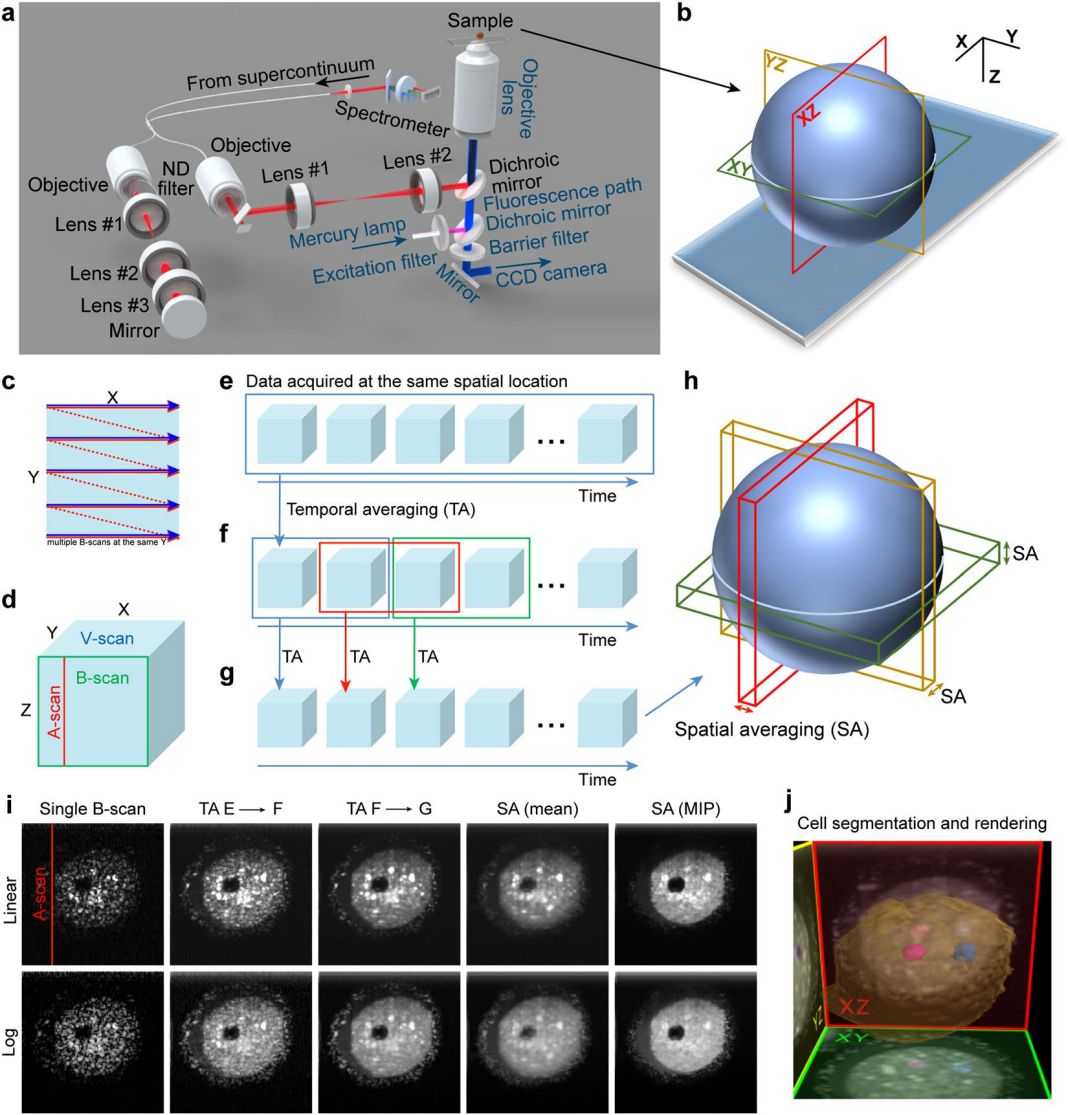
The OCM system and experimental layout. (**a**) Imaging setup: the original parts of the inverted microscope are marked in blue and custom OCM parts in black. (**b**) Schematic of image cross-sections obtained with the OCM setup. (**c**) OCM beam trajectory in the XY plane. Solid line: the beam trajectory during data acquisition. Dotted line: the beam trajectory between Y positions. (**d**) Schematic of the data volume acquired in a single OCM measurement. The A-scan is a single image line obtained from one OCM data acquisition. The B-scan is a single image generated from the A-scans. The V-scan is generated from the B-scans acquired at different Y positions. (**e**–**g**) Stages of the diversified time interval scanning protocol (DTIsp). (**e**) Several V-scans are acquired within ~1 sec and undergo temporal averaging. (**f**) The averaged V-scans from (**e**) were acquired continuously with a time span ranging from ~20–600 sec for periods of tens of hours. (**g**) Temporal averaging (TA) was performed on the averaged V-scans from (**f**) to obtain images with reduced speckle noise. (**h**) Optional spatial averaging (SA) over thick slices of the averaged V-scan. Different types of averaging are possible, including intensity averaging (mean), minimal or maximal intensity projections (MIP). (**i**) Images from consecutive steps of DTIsp processing. Linear intensity mapping allows for a better visualization of the cytoplasmic features, while logarithmic - of the nuclei. (**j**) Cell segmentation and rendering.

In order to visualize live mammalian embryos and oocytes in non-invasive and effective way, we combined an optical coherence microscopy unit built in our laboratory at Nicolaus Copernicus University with an inverted fluorescence microscope (Fig. 1a,b). Using an environmental chamber to ensure appropriate cell culture conditions, we were able to repeat measurements with both devices over an arbitrarily chosen time span and monitor the evolution of the embryo over several hours. The OCT setup utilized the infrared (IR) part of the supercontinuum radiation as a broadband light source to combine sufficient resolution with safety (see the Methods regarding the irradiation energy to which embryos were exposed). The detailed description of the OCM system construction is presented in the Methods. During the OCM examination, the scanning light beam travels over the imaging area following a raster trajectory (Fig. 1c). The sample does not have to move in the z-axis because the distribution of scattering elements along the light beam is recorded in a single data acquisition and processing step^13^ forming a single image line – A-scan (Fig. 1d). A number of A-scans acquired for the same Y coordinate form a single tomogram (B-scan), while a number of B-scans acquired for different Y coordinates form a volume image (V-scan). Single B-scans are affected by strong speckle noise inherent to imaging techniques using illumination with coherent light (visible in the first images of Fig. 1i). Speckle pattern depends on spatial distribution of light scattering elements inside the cell and changes in time due to internal motion of cellular organelles and cytoplasm. Therefore, in order to improve quality of OCM imaging and allow for time-lapse monitoring of cellular processes in embryos, we extended this commonly used 3D OCM imaging scheme and designed the diversified time intervals scanning protocol (DTIsp). In the DTIsp, the beam scans the sample to reconstruct the 3D volume and returns to the same position in the sample several times: after milliseconds (Fig. 1c – multiple B-scans at the same Y coordinate), seconds (Fig. 1e) and tens of seconds (Fig. 1f). Additional spatial compounding, including not only intensity averaging but also minimum or maximum intensity projection (MIP), may be performed (Fig. 1h). This unique approach enables efficient temporal and spatial averaging of the acquired data to reduce the speckle noise and produce high-quality structural images (Fig. 1i, images after spatial and temporal averaging). Finally, OCM images are presented either in linear or logarithmic intensity mapping. Linear mapping emphasizes cellular structures inside the cytoplasm, while logarithmic mapping provides better visualization of nuclear structure. 3D rendering (Fig. 1j), as well as quantitative analysis of obtained data, was performed using custom software (See Methods and Supplementary Materials for more details). More information on DTIsp protocols can be found in Supplementary Materials (Diversified Time Interval Scanning Protocol (DTIsp), Supplementary Fig. S1, Supplementary Table S1, Supplementary Table S2).

**Figure 2:**
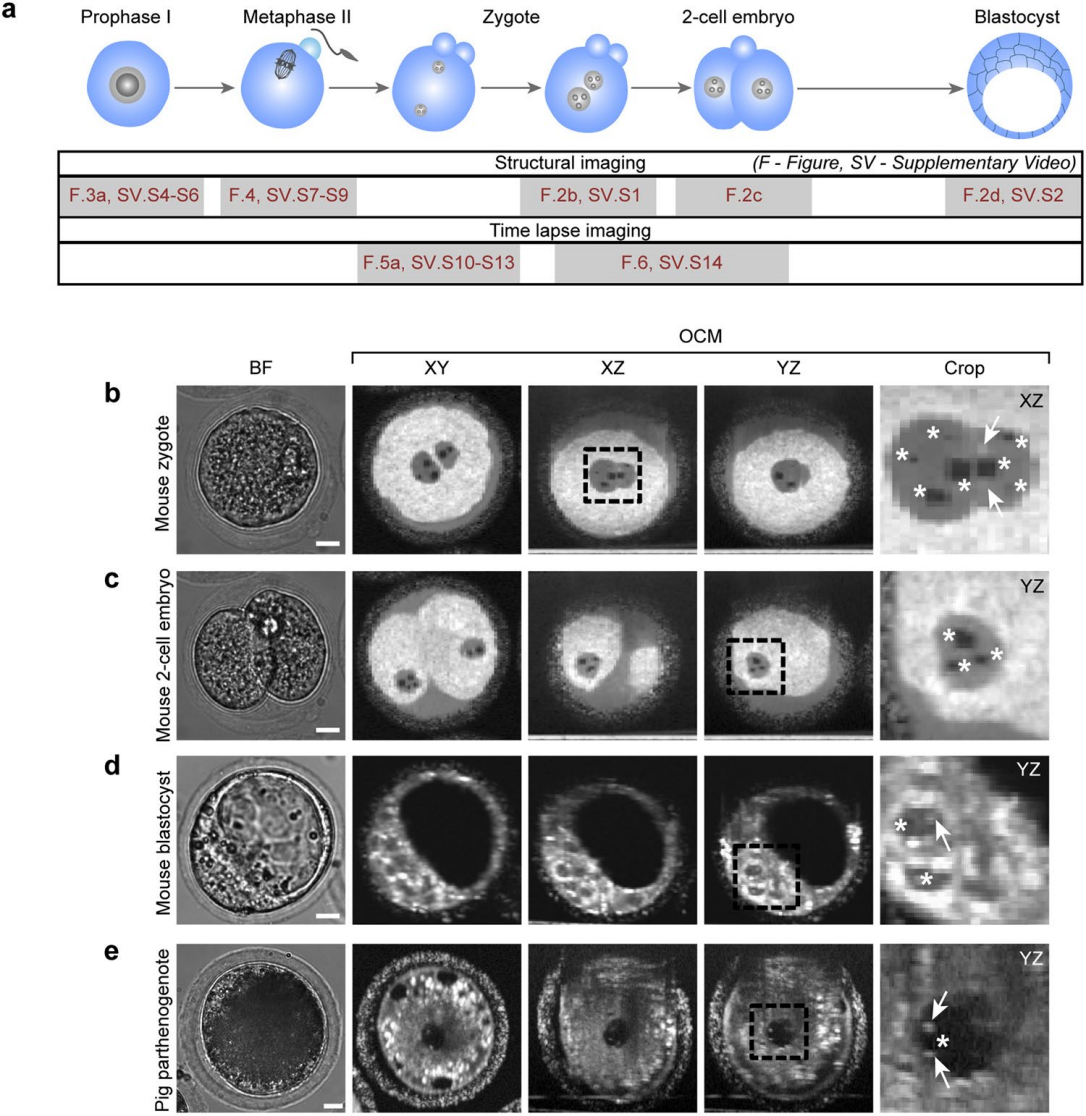
Embryos and their nuclear architecture visualized by OCM. (**a**) Stages in oogenesis and embryonic development subjected to OCM scanning. At some stages only intracellular structures were visualized (structural imaging in a single time-point), in other – both the structures and the dynamics of cellular processes such as pronuclei formation and movement or cell divisions were imaged (time-lapse imaging). (**b–e**) Pronuclei and nucleoli in a mouse zygote (**b**), 2-cell embryo (**c**) and blastocyst (**d**), and in a porcine 1-cell embryo obtained by parthenogenetic activation of an oocyte (**e**). BF – bright field images. OCM images of mouse zygotes and 2-cell embryos (DTIsp #3 protocol) were processed with the minimum intensity projection over 15 μm (Procedure #2). The mouse blastocyst was imaged using DTIsp #3 protocol without spatial averaging (Procedure #1). OCM images of a porcine embryo (DTIsp #2) are shown without spatial averaging (Procedure #1). Asterisks indicate the nucleoli, arrows - specular light reflexes visible in the nuclei that label the nucleoli. Cropped regions are encircled with a dashed line. Scale bars represent 20 μm. See also Supplementary Videos S1–S3 and S14 for more images of these cells.

The main drawback of the proposed approach is a relatively long acquisition of data required for temporal averaging. In the examples shown in this article we averaged ten 3D volumes (acquired in 10 s each) separated by intervals ranging from 22 s to 120 s (see Supplementary Table S1 for details). Therefore, fast cellular processes may blur the averaged image (see discussion in the ‘Dynamics of cellular events’ section). However, it has to be noted that the DTIsp protocols allow for modification of all the intervals between consecutive 3D data acquisitions and adaptation to cellular processes occurring at different timescales, making it a flexible tool to visualize cells at various stages of development (see Fig. 2a).

### Architecture of nuclei

First, we applied this novel live microscopy method to examine the nuclear architecture of mammalian (mouse and pig) oocytes and preimplantation embryos (Fig. 2b–e, Supplementary Videos S1–S3). The 3D OCM data from mouse zygotes and cleaving embryos up to the blastocyst stage were acquired with DTIsp #3 protocol (here and for the rest of the article see Supplementary Table S1 for details on DTIsp protocols) while images of porcine embryos were acquired with DTIsp #2 protocol. The data from mouse zygotes and 2-cell embryos was subsequently processed using a temporal averaging and spatial minimum intensity projection over 15 μm (Procedure #2, here and for the rest of the article see Supplementary Table S2 for details on spatial and temporal averaging). OCM scans of blastocysts were subjected to simple temporal averaging (Procedure #1). The same simple temporal averaging gave particularly spectacular results for porcine embryos: they are filled with lipid droplets that render their cytoplasm non-transparent for standard light microscopy but do not disturb OCM scanning (Fig. 2e, Supplementary Video S3). In OCM scans embryonic nuclei are visible as uniformly grey round shapes, clearly distinguishable from the white speckled cytoplasm. This uniform coloring of nuclei suggests that chromatin is distributed evenly and is optically homogeneous. Smaller, circular blackish shapes present inside the nuclei represent nucleoli, condensed regions build with proteins and nucleic acids responsible for production of rRNA molecules essential for translation. Nucleoli clearly differ in color from the rest of nuclei, which suggests their distinct optical properties. Increased intensity of OCM signal is often visible on the top and bottom of nucleolus border indicating that the surface of nucleolus is very smooth and can specularly reflect light (Fig. 2b–e, Supplementary Videos S1–S3).

Because the number, size and distribution of nucleoli inside zygotic nuclei are the morphological criteria used to assess the quality of mammalian embryos^1, 14–16^, our imaging method may facilitate the selection of the most viable embryos, e.g. human embryos suitable for transfer to patients. Moreover, the visualization of nuclei in unlabeled, multicellular embryos, such as blastocysts, may provide a simple method for calculating cell numbers, either in the whole embryo or in its inner cell mass (ICM). This technique for counting cell numbers could be a significantly more objective criterion for blastocyst quality than the simple assessment of the area and shape of the ICM that is currently used in medical and veterinary practice^1^. Nuclear architecture of unlabeled cells can be also visualized by harmonic generation microscopy, however it typically provides only a limited number of z planes (approximately 7–10 compared to 70–100 pixels along z-axis in OCM tomograms where the cell is visible). Moreover, the total irradiation energy to which sample is exposed in this technique in a single measurement is approximately 6 times higher than in OCM system reported here^17, 18^.

### Chromatin structure

**Figure 3:**
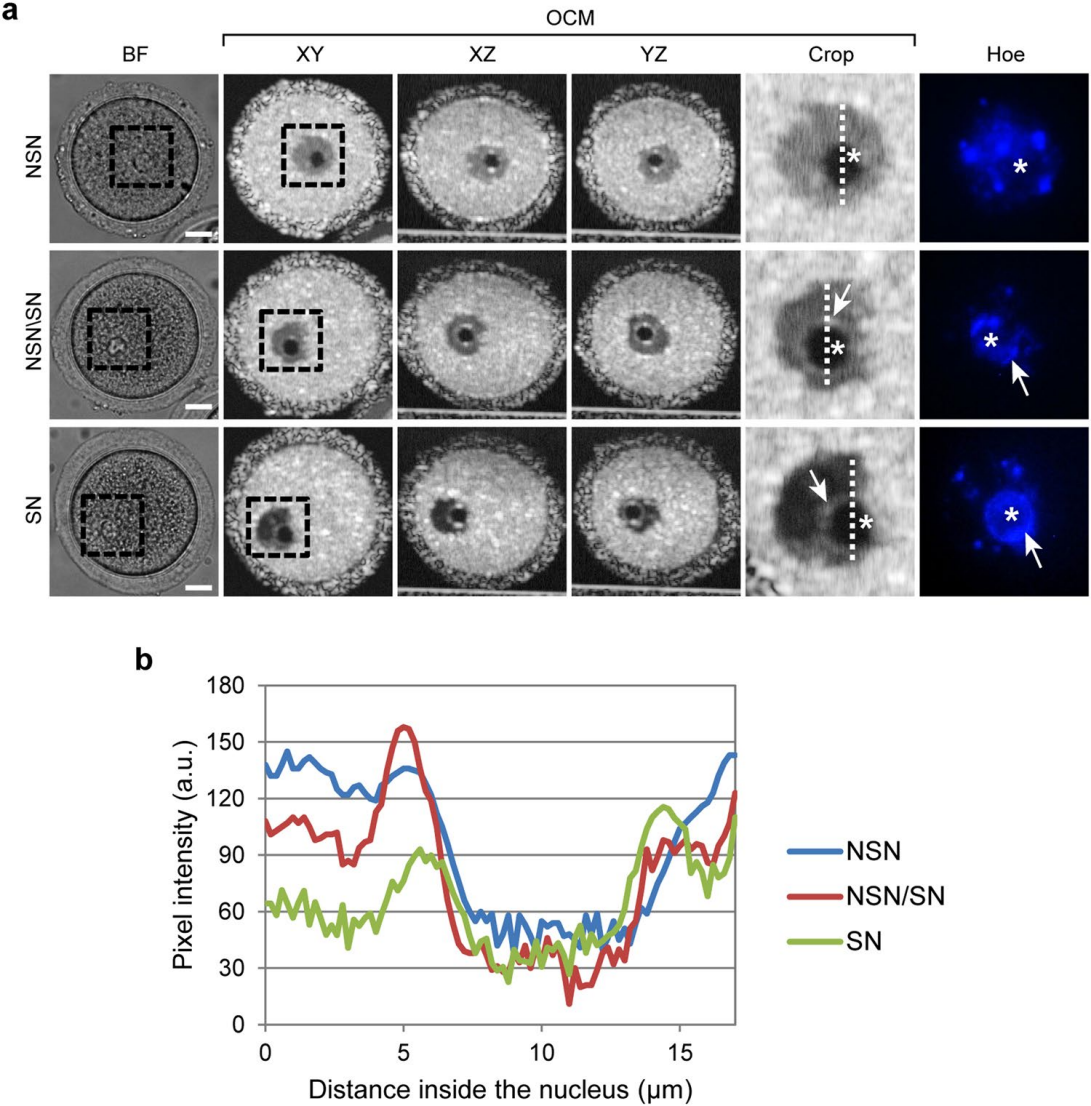
Chromatin structure in mouse oocytes visualized by OCM. (**a**) Chromatin structures observed in prophase I mouse oocytes. The panels show bright field images (BF), fluorescent images of DNA stained with Hoechst 33342 (Hoe) and OCM images (DTIsp #1) without spatial averaging (Procedure #1). NSN – oocyte in the non-surrounded nucleoli (NSN) stage (transcriptionally active); SN – oocyte in the surrounded nucleoli (SN) stage (transcriptionally silent); NSN/SN – oocyte in transition between the NSN and SN stages. Asterisks mark nucleoli, arrows - condensed chromatin around nucleoli. Cropped regions are encircled with a dashed line. (**b**) Profile plots of pixel intensity measured along nuclear perimeter marked with a dashed line in cropped images in (**a**). Scale bars represent 20 μm. See also Supplementary Videos S4–S6 for more images of these cells.

The OCM scans (DTIsp #1) subjected to averaging over time (Procedure #1) were also used to distinguish different types of chromatin condensation, e.g. in nuclei of unlabeled oocytes arrested in the prophase of the 1^st^ meiotic division (ProI). Depending on their transcriptional status, mammalian ProI oocytes display different chromatin distribution patterns. The nuclei of transcriptionally active oocytes contain chromatin that is uniformly dispersed around the nucleoli (in a mouse model: NSN – oocytes with non-surrounded nucleoli); however, in oocytes in which transcription has already ceased, chromatin condenses to form a ring around the nucleoli (SN – oocytes with a surrounded nucleoli)^2, 19^. Importantly, SN oocytes display significantly higher developmental potential than NSN oocytes, as judged by their ability to complete meiosis and form a blastocyst after fertilization^20–23^. In the OCM images the inside of an NSN nucleus is uniformly gray, except for a black nucleolus: it means that the chromatin is uniformly distributed around the nucleolus and is optically homogeneous. In contrast, the chromatin structure in an SN nucleus is more complex in the OCM scan; black and gray regions indicate that the chromatin is distributed unevenly and forms a ring around the nucleolus. In oocytes that are in transition between the NSN and SN states, the characteristics of the SN and NSN OCM images are combined: a bright gray ring surrounding the nucleolus is visible on the dark gray background that fills the rest of the nucleus (Fig. 3a, Supplementary Videos S4–S6). We confirmed that the OCM images indeed reflect chromatin structure by fluorescent staining of the DNA (Fig. 3a). Additionally, a quantitative analysis of the pixel intensity profile of the OCM scans, performed along the nuclear diameters, confirmed that each type of nuclei has indeed different distribution of bright and dark intranuclear regions (i.e. differently condensed chromatin) (Fig. 3b).

Until now, the assessment of chromatin distribution in live cells, e.g. ProI oocytes, has only been conducted using fluorescence or confocal microscopy and required either transgenic cell lines expressing fluorescently tagged chromatin proteins, such as histones, or fluorescent DNA markers that severely impair cell viability. The OCM approach represents a non-invasive procedure that does not require application of any harmful dyes to non-transgenic cells; therefore, it may be used in medical and veterinary practice to select ProI oocytes with higher developmental potential. Indeed, the demand for this type of selection method has increased because IVF clinics and animal breeding facilities more and more frequently use oocytes isolated from ovaries (i.e. in ProI) and matured *in vitro* ^24–26^. It is possible that oocytes with a different chromatin distribution, and, in consequence, a different transcriptional status, could be distinguished by a simple measurement of the mean pixel intensity inside the nucleus. Our preliminary results indicate that it is higher in NSN than in SN oocytes (data not shown).

### Spindle structure

**Figure 4:**
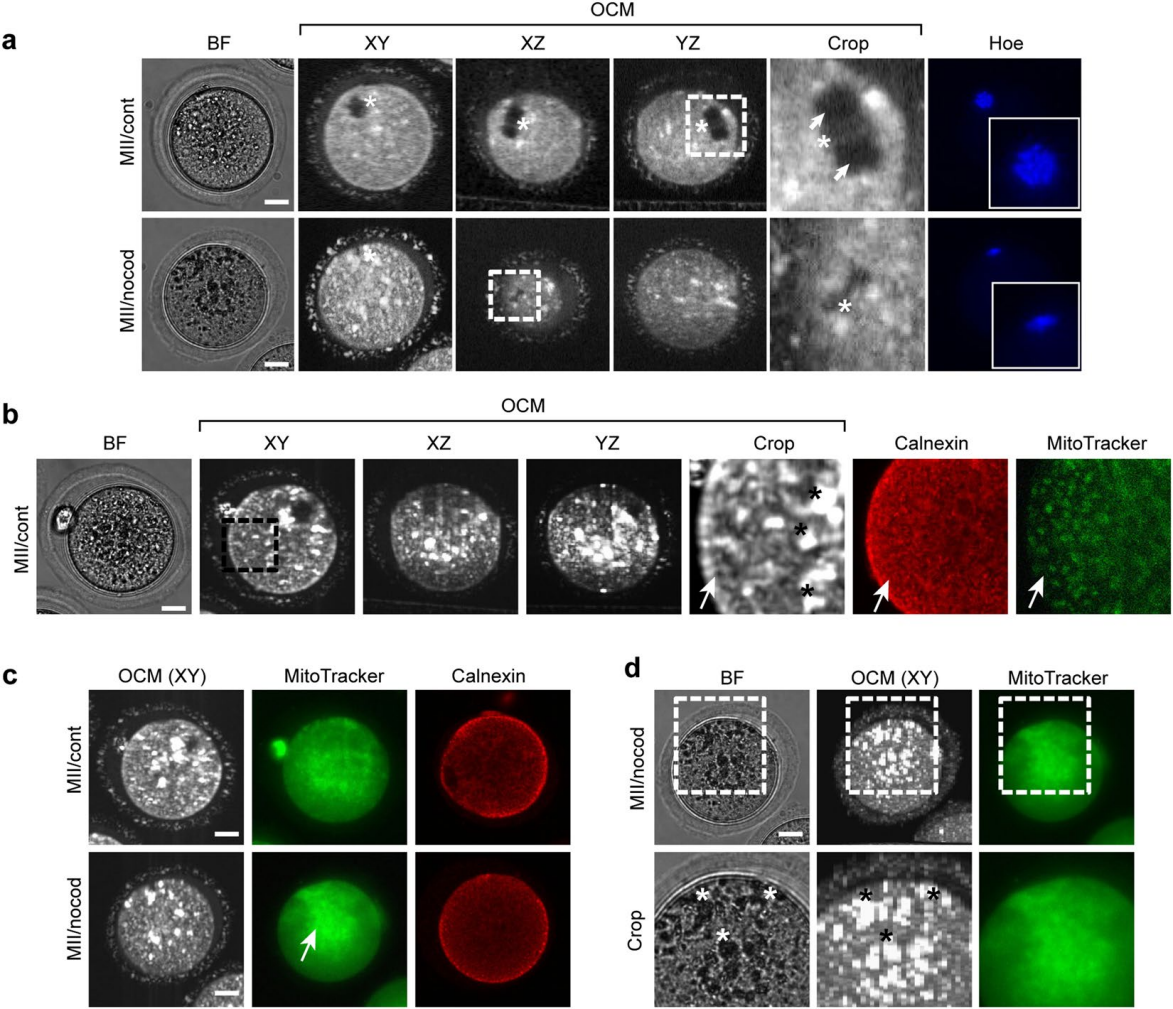
Metaphase spindle and cytoplasmic organelles in ovulated mouse oocytes visualized by OCM. (**a**) A metaphase II spindle in control (MII/cont) or nocodazole-treated (MII/nocod) ovulated metaphase II mouse oocytes. The panels consist of bright field images (BF), fluorescent images of DNA stained with Hoechst 33342 (Hoe) and OCM images. Asterisks indicate chromosomes, arrows - microtubular part of the metaphase spindle. Cropped regions are encircled with a dashed line. (**b**) Membranous organelles (probably endoplasmic reticulum (ER) cisterns and mitochondria) in metaphase II mouse oocytes. The panels show bright field images (BF), OCM images, fluorescent images of ER cisterns stained with an anti-calnexin antibody (in red) and mitochondria stained with MitoTracker Green (in green). Arrows mark fine network of membranous cisterns inside the oocyte, asterisks - big white patches representing probably lipid droplets. The cropped OCM region is encircled with a dashed line. (**c**) Membranous organelles in control (MII/cont) or nocodazole-treated (MII/nocod) metaphase II mouse oocytes. The panels show OCM images, mitochondria stained with MitoTracker Green (in green; the same oocytes as for the OCM), and fluorescent images of ER cisterns stained with an anti-calnexin antibody (in red). Arrow indicates central accumulation of mitochondria in nocodazole-treated oocytes. (**d**) Membranous organelles in nocodazole-treated metaphase II mouse oocytes. Distribution of big white OCM patches (probably lipid droplets) reflects localization of dark granules (BF), but not localization of fluorescently stained mitochondria (MitoTracker). Asterisks mark dark granules (in BF) and corresponding white patches (in OCM). Cropped regions are encircled with a dashed line. OCM images were acquired with DTIsp #3 protocol. OCM images in (**a–c**) are presented without spatial averaging (Procedure #1) while OCM images in (**d**) is spatially averaged over 70 μm (Procedure #3). Scale bars represent 20 μm. See also Supplementary Videos S7– 10 for more images of these cells.

OCM structural imaging (DTIsp #3 followed by Procedure #1) also provides information about the shape and structure of the metaphase spindle with clearly distinguishable chromosomes. Part of the spindle that is built with microtubule fibers is visible in the OCM scan as a dark gray region, whereas centrally located chromosomes are clearly brighter in color, confirming different optical properties of these two spindle components (Fig. 4a, Supplementary Videos S7 and S8). The characteristic barrel shape of the metaphase II spindle disappeared when oocytes were treated with nocodazole, a drug that depolymerizes microtubules (Fig. 4a, Supplementary Video S9).

The shape of the metaphase spindle and the arrangement of chromosomes are also indicators of the oocyte quality. An abnormal structure of the spindle and/or chromosomal plate usually results in the faulty segregation of the genetic material and aneuploidies^3, 27^. The great advantage of OCM visualization over polarization microscopy, another non-invasive method for imaging the spindle^27, 28^, is that OCM allows for a detailed analysis of the spindle shape, independent of its orientation to the focal plane; even if the spindle is perpendicular to the XY plane, its shape will be well depicted in the XZ and YZ planes (Fig. 4a).

### Networks of membranous organelles

OCM scanning (DTIsp #3 followed by Procedure #1) also allowed us to visualize the intracellular organelle network (Fig. 4b). Comparison between OCM images and organelle-specific fluorescent stainings indicates that the fine web of white interconnected speckles clearly visible in OCM scans most likely represents imposition of ER cisterns and mitochondria. The pattern of OCM signal resembles very well the fluorescently labeled ER network, particularly in the cortical region of the oocytes. Participation of mitochondria in the OCM signal cannot be excluded, although their distribution pattern differs from the pattern of OCM speckles and therefore definitely they are not the only source of the cytoplasmic OCM signal (Fig. 4b). The big white patches imposed on the finer web of speckles represent probably lipid droplets that are often visible in mouse oocytes and embryos^17, 18^.

Network of membranous organelles, although much less defined than that recorded by our OCM system, was also visualized by a full-field OCM method^12^. In this paper authors proposed that the cytoplasmic signal came mostly from mitochondria. However, the same as in our present study, closer inspection of their images show certain differences between the OCM signal and the distribution of these organelles. In order to determine whether the fine web of OCM cytoplasmic speckles is generated predominantly by ER or mitochondria, we investigated how nocodazole affects distribution of these two types of organelles and the OCM cytoplasmic signal. Nocodazole-induced depolimerization of microtubules led to translocation of mitochondria towards the cell center, but altered neither ER nor fine OCM speckle distribution (Fig. 4c). Moreover, we noticed that nocodazole triggered cytoplasmic accumulation of big white patches of the OCM signal (i.e. presumed lipid droplets), which mirrored the distribution of dark granules visible in the corresponding bright field images. However, neither distribution of the white patches, nor localization of the dark granules reflected precisely the pattern of mitochondria (Fig. 4d). Taken together, these observations indicate that the fine OCM speckle network most likely represent mainly ER cisterns (although mitochondria participation is also possible), whereas big white OCM patches – dark cytoplasmic granules (possibly lipid droplets).

Similarly to the nuclear structures described in previous paragraphs, the localization of cytoplasmic organelles serves as another marker of oocyte and embryo quality in clinical or veterinary practice^1, 29^. For example, human oocytes displaying a moderate cortical ring of clear, organelle-free cytoplasm have been reported to have a significantly higher developmental potential, as assessed by the pregnancy rate, than oocytes with no or too extensive cortical ‘halos’^29, 30^. As we showed here, OCM scanning enables non-invasive and accurate analysis of the distribution of the cytoplasmic components.

### Dynamics of cellular events

**Figure 5:**
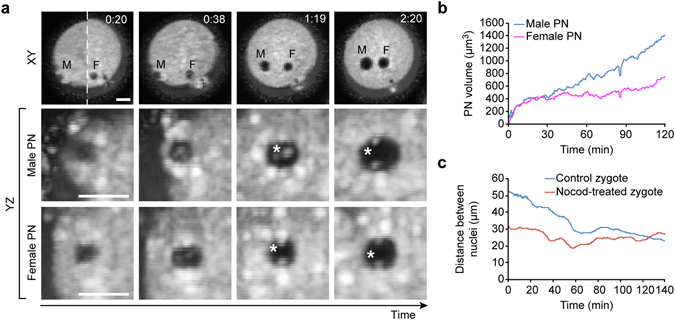
Formation and movement of zygotic nuclei visualized by OCM. (**a**) Formation of male (M) and female (F) pronuclei in mouse zygotes. OCM images (XY plane) selected from the time-lapse recording obtained with DTIsp #4 protocol are presented using minimum intensity projection of 15 μm-thick slices (Procedure #2). Pronuclei cropped in YZ plane from the same time points as the XY projections are shown without intensity averaging (Procedure #1) and reveal formation of nucleoli (marked with asterisks). (**b**) Changes in the pronuclei volume were measured with OCM for 2 hrs, with 30 sec intervals between the acquisition of each 3D dataset. Each 3D dataset was automatically segmented using custom algorithms to extract the voxels belonging to pronuclei and to calculate the volume and position of each pronucleus. A gradual increase in volume of both female and male pronucleus is clearly visible. (**c**) Changes in the distance between pronuclei recorded in control and nocodazole-treated zygotes. Zygotes were filmed for 2.5 hrs, with 30 sec intervals between the acquisition of each 3D dataset. Recordings started at approximately the same moment after fertilization, as judged by morphological markers of the post-fertilization events (i.e. presence of the fertilization cone – a protrusion above the sperm chromatin, and formation of the second polar body). The initial position of nuclei in control and nocodazole-treated zygotes was different (the initial distances were 50 and 30 µm respectively) due to the different sperm entry-point. In control zygotes the distance between pronuclei clearly decreases over time, whereas in nocodazole-treated zygotes it stays the same. Scale bars represent 20 µm. See also Supplementary Videos 11–14 for more images of these cells.

Because our OCM system provides not only high-quality structural data but also records events over time, we applied it (using DTIsp #4) to monitor cellular processes, such as formation and translocation of pronuclei in a zygote (Fig. 5a). Because nuclei were clearly visible as dark shapes against the bright background of the cytoplasm, they were automatically segmented (see the Supplementary Materials for detailed description of the cell segmentation algorithms). This approach allowed us to perform a detailed analysis of pronuclear development, i.e. to determine when exactly pronuclei are formed, and to calculate their volume, velocity and direction of movement over time (Fig. 5b, Supplementary Videos 11 and S11). To exemplify the usefulness of our OCM method in dynamic measurements, we examined whether OCM scanning detects the difference in pronuclei movement in control and nocodazole-treated zygotes. Nocodazole blocks microtubule-dependent pronuclear translocation towards each other and to the cell center^31^. Indeed, our measurements clearly showed a significant and expected difference in the pronuclei trajectories and distance traveled in the nocodazole-treated zygotes compared with the control zygotes (Fig. 5c, Supplementary Videos S12 and S13).

**Figure 6:**
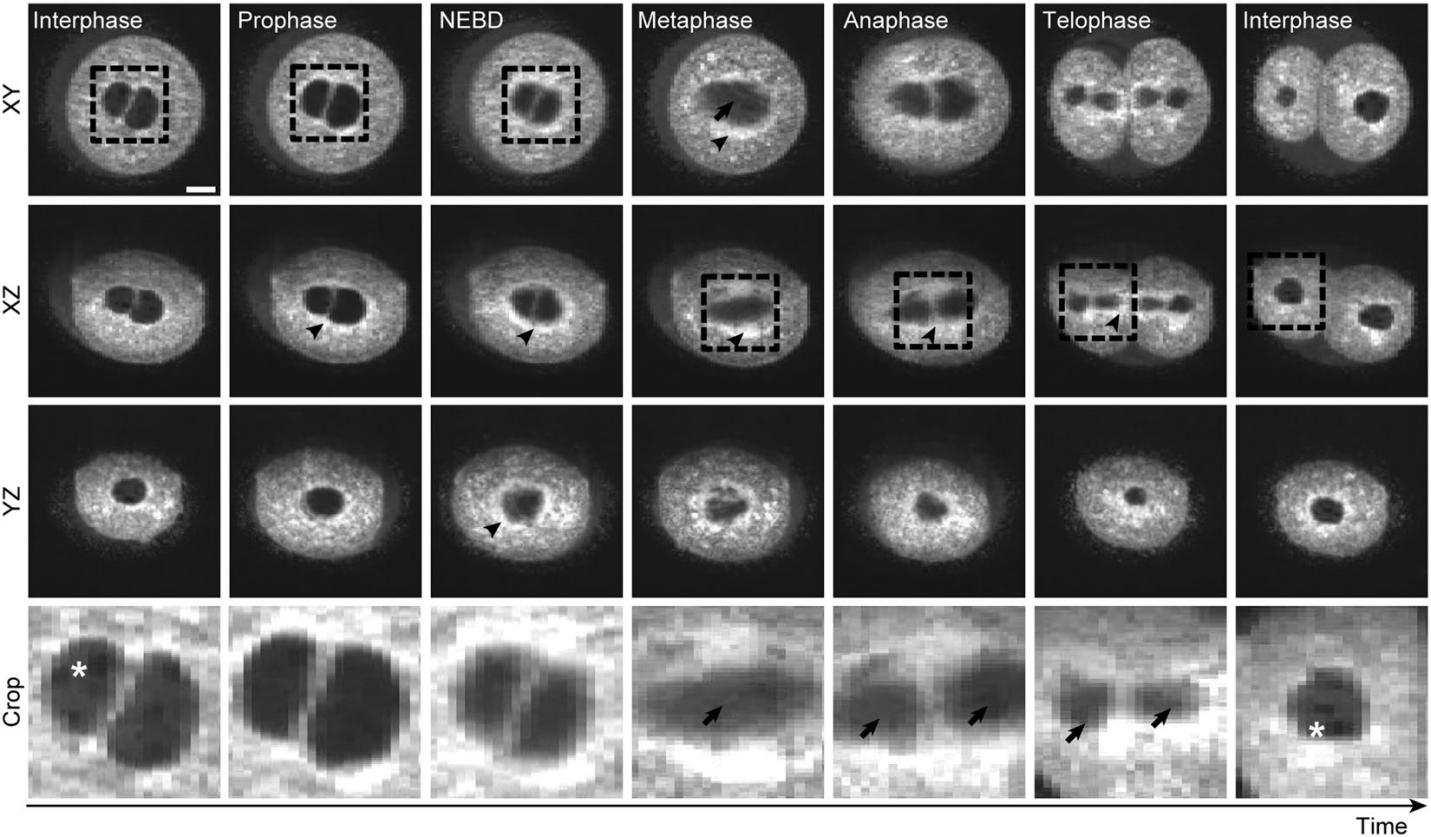
The first embryonic division visualized by OCM. Mouse zygotes were subjected to OCM time-lapse imaging DTIsp #5 protocol for 13.5 hrs with 120 s intervals between the acquisition of each 3D dataset. Panels present OCM minimum intensity projections of 15 μm thick slices (Procedure #2) selected from the time-lapse recording, showing main events during the first embryonic division: disappearance of nucleoli (in prophase), nuclear envelope break-down (NEBD), formation of the metaphase spindle (metaphase), segregation of chromosomes (anaphase, telophase) and formation of new nucleus with nucleoli (interphase - the last panel). Asterisks indicate the nucleoli, arrows - the microtubular parts of the spindle, arrowheads - a ring formed by membranous organelles (probably ER and mitochondria) around the nuclear apparatus and the spindle during the cell division. Cropped regions are encircled with a dashed line. Chromosomes are not visible with this type of spatial averaging. Scale bars represent 20 µm. See also Supplementary Video S14 for more images of these cells.

Next, we applied OCM (DTIsp #5) to follow the first embryonic division (Fig. 6, Supplementary Video S14). During 13 hour-long recording we were able to visualize in 3D events such as disappearance of the nucleoli and pronuclei, formation of the mitotic spindle and reorganization of the ER and mitochondria network accompanying the embryonic division. We also captured re-establishment of the nuclei and nucleoli in the divided 2-cell embryo, proving that the OCM is also useful for long-term dynamic studies. In this scanning protocol we acquire 3D data sets every 120 s, which made temporal averaging more challenging. We noticed that after temporal averaging OCM images showing the last phase of cellular division (telophase) became slightly blurred, but it did not affected significantly the general quality of imaging or the automatic segmentation of the nuclear apparatus: the key intracellular structures were still well visible (see Supplementary Video S14, frames 255–280).

As dynamics of the first embryonic divisions is an important biomarker of embryonic quality^1^, information provided by OCM may be useful in embryo assessment protocols either in a clinical setting or veterinary practice. It can be also an interesting addition to the imaging toolkit used in basic biological research, e.g. when the mechanisms of nuclear movement, formation and/or break-down are examined.

In summary, the OCM system combined with optimized data acquisition protocols and specialized image processing algorithms enables the non-invasive 4D imaging of intracellular processes and architecture with clarity that is comparable in many respects to the images obtained with confocal microscopy. In contrast to confocal microscopy, however, it does not require any cell labeling or pre-processing. This unique feature makes OCM widely applicable, particularly in a clinical setting. A quick OCM scan provides substantial information about the chromatin structure, shape of a metaphase spindle and distribution of the intracellular network of organelles, such as ER cisterns and mitochondria. Most importantly, the OCM system can also follow intracellular events over a prolonged time by monitoring the dynamic changes occurring in the examined cells. All of this information is used as an important outcome for functional cellular studies and as an embryo quality predictor in IVF protocols. Therefore, we believe that our OCM system may represent a new chapter in non-invasive, dye-independent 4D imaging at intracellular resolution.

## Methods

### Optical Coherence Microscopy (OCM)

The fiber-based OCM setup utilized a supercontinuum light source (SuperK EXTREME EXW-4, NKT Photonics A/S, Danmark). In this particular study, a spectrum at an 800 nm central wavelength with −3 dB spectral bandwidth of 115 nm was used. The OCM interferometer sample arm shared an imaging objective (Nikon Plan Fluor 20x, NA = 0.5) with an inverted microscope system. Power in the reference arm was adjusted with a neutral density filter (NDF). A pair of prisms (BK7 optical glass) compensated for the difference in dispersion between the interferometer arms. The sample was illuminated at an average optical power of 1.2 mW. The sensitivity measured at a 20 µs acquisition time was ~88–89 dB. The axial resolution was 1.9 µm in the tissue. The lateral resolution tested using the USAF resolution target was 1.9 µm. Interferometric fringes of 2048 pixels were captured using a line-scan CMOS camera with 140 kHz line rate (Basler Sprint spL2048-140 km, Basler AG, Germany) and acquired with a high-bandwidth framegrabber (PCIe-1433, National Instruments, USA), resulting in a 0.9 mm imaging range. Standard steps of OCT data processing were performed to create a tomogram line from the acquired spectrum and included fixed pattern noise removal, resampling to the wavenumber domain, residual dispersion correction and spectral shaping followed by Fourier transformation^32^. The OCM beam scanned the sample in both lateral directions using a pair of galvanometric scanners (8320 K, Cambridge Technology Inc, USA) driven by an analog I/O card (PCI-6733, National Instruments, USA). Custom-designed software (C++/C#) was used to synchronize and operate all components of the system.

### Inverted Fluorescence Microscopy (IFM)

We used an inverted microscope (Eclipse Ti-E, Nikon, Japan) equipped with a monochromatic camera (DS-Qi1Mc, Nikon, Japan) connected to a right-side microscope port with 100% transmission. Bright field imaging was performed with a halogen lamp and Nikon Plan Fluor objective (20x, NA = 0.5). For fluorescence imaging, a mercury lamp with a filter set (an excitation filter, a dichroic mirror and a barrier filter: DAPI-1160B filter set (Semrock Inc., USA) for Hoechst 33342 staining and FITC-5050A filter set (Semrock Inc., USA) for MitoTracker Green staining) were used. An XY motorized microscope stage (H117, Prior Scientific Inc., USA) was equipped with an incubator system (OKOLAB S.R.L., USA) to maintain stable conditions and cell viability (37.5 °C throughout the experiments).

### Combined OCM and IFM Imaging

A custom-designed coupling adapter allowed for a precise alignment of the optical axes of the OCM and IFM systems. The OCM beam entered the optical path of the microscope through a dichroic mirror (transmission in the range of 400–700 nm, reflection >700 nm). The XY coverage of both systems was carefully aligned. During prolonged recordings, we turned off IFM illumination at set time intervals between consecutive measurements. Likewise, the OCM beam was deflected off the optical axis of the system after each OCM measurement. As a result, the sample illumination was substantially reduced during prolonged imaging. Each OCM measurement was recorded shortly after the IFM acquired a bright field image. Therefore, we were able to compare the results obtained with both systems at every time point during the prolonged measurements.

### Preparation of Oocytes and Embryos

All animal studies were approved by the Local Ethics Committee for Experimentation on Animals no. 1 (Warsaw, Poland), designated by the National Ethics Committee for Experimentation on Animals (Poland), and were performed in compliance with the national regulations.

Mouse oocytes and embryos were isolated from 2- to 3-month-old F1 (C57BL/6/Tar x CBA/Tar) females that were euthanized by cervical dislocation. To obtain embryos and ovulated oocytes, females were superovulated with an intraperitoneal injection of 10 IU of pregnant mare serum gonadotrophin (PMSG, Intervet, Netherlands) followed by an injection of 10 IU of human chorionic gonadotrophin (hCG, Intervet, Netherlands) 48 hrs later and, when embryos were required, mating with 6- to 10-month-old F1 males. Next, ovulated oocytes and zygotes were recovered from the oviducts 16–22 hrs after the hCG injection and immediately placed in a hyaluronidase solution (150 IU/ml, Sigma-Aldrich, Poland) to remove the cumulus cells. Blastocysts were flushed from the uterus into M2 medium (M16 medium buffered with HEPES) 90 hrs after the hCG injection. Prophase I oocytes were recovered from the ovaries of F1 females that had been primed with 10 IU of PMSG 48 hrs after injection. These oocytes were isolated from fully grown ovarian follicles and placed in the M2 medium.

Porcine embryos were obtained by the parthenogenetic activation of *in vitro* matured porcine oocytes. The cumulus-oocyte complexes were collected from the ovaries of commercially slaughtered 5–6-month-old gilts weighing 100–110 kg. *In vitro* maturation was performed in basic NCSU23 medium supplemented with 10% (v/v) porcine follicular fluid, 10 U/ml hCG (Intervet, Netherlands), 10 U/ml PMSG (Intervet, Netherlands), 0.1 mg/ml cysteine and 50 μg/μl gentamicin sulfate, as previously described^33^. Mature oocytes that had been freed from cumulus cells were activated with 5 μM ionomycin in TALP medium for 5 min in the dark, washed in NCSU23 medium supplemented with 4 mg/ml BSA and transferred to fresh NCSU23 supplemented with 2 mM 6-DMAP (6-Dimethylaminopurine, Sigma-Aldrich, Poland) for 4 hrs in a 5% CO_2_, and 5% O_2_ atmosphere at 39 °C. Next, activated oocytes were transferred to drops of NCSU23 medium with 4 mg/ml BSA for further culture under the conditions described above.

For imaging, oocytes and embryos were transferred to a 35 mm plastic dish with a 0.16 to 0.19 mm-thick glass bottom (MatTek, USA) filled with M2 (in the case of mouse oocytes and embryos) or TALP medium (in the case of porcine embryos). The glass bottom was coated with a thin (~100–150 μm) layer of agar (1% solution in 0.9% NaCl, Bacto-agar, BD, USA) to avoid the unwanted, strong OCM signal from the glass surface. The medium was covered with a layer of mineral oil (Sigma-Aldrich, Poland) to prevent evaporation. In some experiments, the oocytes and embryos were pre-treated with 5 µg/ml nocodazole (Sigma-Aldrich, Poland) for 1 hour and then filmed in M2 medium supplemented with nocodazole (at the same concentration).

### Fluorescence Staining

Live oocytes and embryos were incubated with 100 ng/ml Hoechst 33342 (Sigma-Aldrich, Poland) and 200 nM MitoTracker Green (MolecularProbes, ThermoFisher Scientific, USA) to visualize the DNA and mitochondria. To visualize the ER, the oocytes were fixed with 4% paraformaldehyde (ThermoFisher Scientific, USA, 30 min, RT), permeabilized with 0.2% Triton X-100 (Sigma-Aldrich, Poland, 25 min at RT) and blocked in 3% bovine serum albumin (Sigma-Aldrich, Poland, overnight at 4 °C). Next, the oocytes were incubated with a primary rabbit polyclonal anti-calnexin (an ER marker) antibody (Abcam, UK, 1:200, overnight at 4 °C), briefly washed in PBS and 3% BSA and incubated with a secondary Alexa Fluor 633-conjugated goat anti-rabbit IgG antibody (ThermoFisher Scientific, USA, 1:200, 2 hrs at RT). The oocytes and embryos were imaged using the IFM system (Eclipse Ti-E, Nikon, USA) or a confocal microscope (Olympus and Zeiss, Germany).

### OCM Sample Illumination – Risk Assessment

The power density in a focal plane of the OCM system was 1.27 $\ast$ 10^4^ W/cm^2^ (1.2 mW and a resolution of 1.9 μm), which was 3 to 7 orders of magnitude lower than the density in confocal or multi-photon microscopy^9^. König *et al*. investigated the cloning efficiency of Chinese hamster ovary cells using an 800 nm femtosecond laser^34^. The safe level for 100% cloning efficiency was measured at power levels ≤1 mW. The illumination power in our OCM system was slightly higher (1.2 mW); however, the potential impact on the sample was significantly reduced by the pulse width, which was in the nanosecond scale for the supercontinuum source used here. Moreover, in our experiments, the OCM beam continuously scanned the sample; therefore, the illumination time for a single point on the sample was only a small fraction of the total measurement time. For the main DTIsp protocol used in the study for cell evolution monitoring (DTIsp #4, see Supplementary Table S1 for details on DTIsp protocols) the total illumination time for a single point on the sample during the measurement session lasting 2.5 hrs was approximately 120 ms with total energy delivered to the cell equal 3 J. For DTIsp #5 it was 160 ms single point illumination and 4 J total energy delivered during 13.5 h. One of the indicators that our OCM measurements did not harm the cells was the observation of the first embryonic division.

### Automated Tracking of Zygote Dynamics

We devised a dedicated and almost entirely automatic image analysis algorithm to segment the cellular structures. First, a human expert marks the locations of both the male and female pronuclei in a single image (frame) in the sequence of 3D frames. Starting from that frame, the algorithm traces the cellular structures forward and backward in time in each frame by first extracting the entire cell body, then detecting the pronuclei, spindles and nuclei, and concluding with the segmentation of the nucleoli. Extraction of the cell body involves thresholding individual z-layers using Otsu’s method^35^ with a locally adjusted threshold, filling all apparent cavities (holes) that may result from that process, morphological opening to delineate individual objects, marking the largest resulting object as the cell body, and finally filling all apparent cavities in the cell body (e.g. male pronucleus close to cell membrane). This last step also marks the locations of the pronuclei in the previous frame, when that information is available. We then proceeded to detect the pronuclei, spindles or nuclei (depending on the phase of embryonic development), which involves Otsu’s thresholding with a dynamically tuned threshold, followed by approximation of the resulting objects with small ellipsoids and using those ellipsoids as starting points for independent watershed segmentations. The holes in the segmented objects were then filled, and the objects that did not appear in the previous frame were rejected. This part of the algorithm also assessed the merging of the pronuclei (using Otsu’s thresholding with two thresholds^36^) and nuclear division (by detecting sudden shape changes). We independently examined each pronucleus or nucleus and applied adaptive thresholding combined with morphological closing to merge the adjacent structures and detect the nucleoli. The complete algorithm is more sophisticated and involves additional elements that are described in the Supplementary Materials.

### Data availability statement

The datasets generated and/or analyzed during the current study are available from the corresponding author on reasonable request.

## Electronic supplementary material

Supplementary Materials

Supplementary Video S1. Mouse zygote with two pronuclei

Supplementary Video S2. Mouse blastocyst.

Supplementary Video S3. Porcine parthenogenote with a single pronucleus.

Supplementary Video S4. Mouse prophase I oocyte at the non-surrounded nucleoli (NSN) stage.

Supplementary Video S5. Mouse prophase I oocyte in transition from the non-surrounded nucleoli (NSN) stage to the surrounded nucleoli (SN) stage.

Supplementary Video S6. Mouse prophase I oocyte at the surrounded nucleoli (SN) stage.

Supplementary Video S7. Mouse metaphase II oocyte with a spindle placed perpendicularly to the XY plane.

Supplementary Video S8. Mouse metaphase II oocyte with a spindle placed parallel to the XY plane.

Supplementary Video S9. Nocodazole-treated metaphase II oocyte that lacks a metaphase spindle.

Supplementary Video S10. Formation and movement of pronuclei in a mouse zygote subjected to time-lapse imaging.

Supplementary Video S11. 3D visualization of pronuclear trajectories in a mouse zygote tracked using our custom algorithm.

Supplementary Video S12. Formation and movement of pronuclei in a nocodazole-treated mouse zygote subjected to time-lapse.

Supplementary Video S13. 3D visualization of pronuclear trajectories in a nocodazole-treated mouse zygote tracked using our custom algorithm.

Supplementary Video S14. Mouse embryo at 1- to 2-cell transition subjected to a time-lapse imaging.
