# Supplementary material for: Optical coherence microscopy as a novel, non-invasive method for the 4D live imaging of early mammalian embryos
Source: Sci Rep. 2017 Jun 23;7:4165. doi: 10.1038/s41598-017-04220-8 (PMC5482811; doi:10.1038/s41598-017-04220-8)
Supplement: Supplementary file 1 — Supplementary Materials [file 41598_2017_4220_MOESM1_ESM.pdf]

Supplementary materials:

Optical coherence microscopy as a novel, non-invasive method for the 4D live imaging of early mammalian embryos

Karol Karnowski[1], Anna Ajduk[2,*], Bartosz Wieloch[3], Szymon Tamborski[1], Krzysztof Krawiec[3], Maciej Wojtkowski[1,4] & Maciej Szkulmowski[1,*]

[1]Institute of Physics, Faculty of Physics, Astronomy and Informatics, Nicolaus Copernicus University, Grudziadzka 5, 87-100 Torun, Poland, [2]Department of Embryology, Faculty of Biology, University of Warsaw, Miecznikowa 1, 02-096 Warsaw, Poland, [3]Institute of Computing Science, Poznan University of Technology, Piotrowo 2, 60-965 Poznan, Poland, [4] Institute of Physical Chemistry, Polish Academy of Sciences, Kasprzaka 44/52, 01-224 Warsaw, Poland

* Corresponding authors: aajduk@biol.uw.edu.pl (AA), maciej.szkulmowski@fizyka.umk.pl (MS)

Supplementary Videos captions

Supplementary Video S1. Mouse zygote with two pronuclei. **(A)** Bright field. **(B, D, E)** OCM. OCM data obtained with DTIsp #3 resampled to 240x240x240 voxels; consecutive slices in the XY **(B)**, XZ **(D)** and YZ **(E)** planes are shown. **(C)** 3D visualization of automatic image segmentation of the OCM image, with the female pronucleus marked in blue, the male pronucleus marked in red, and the nucleoli marked in yellow.

Supplementary Video S2. Mouse blastocyst. (A) Bright field. **(B, C, D)** OCM. OCM data obtained with DTIsp #3 resampled to 240x240x240 voxels; consecutive slices in the XY **(B)**, XZ **(C)** and YZ **(D)** planes are shown.

Supplementary Video S3. Porcine parthenogenote with a single pronucleus. (A) Bright field. **(B, C, D)** OCM. OCM data obtained with DTIsp #2 resampled to 240x240x240 voxels; consecutive slices in the XY **(B)**, XZ **(C)** and YZ **(D)** planes are shown.

Supplementary Video S4. Mouse prophase I oocyte at the non-surrounded nucleoli (NSN) stage. (A) Bright field. **(B)** Hoechst 33342-stained chromatin visualized with fluorescence microscopy. **(C, E, F)** OCM. OCM data obtained with DTIsp #1 resampled to 240x240x240 voxels; consecutive slices in the XY **(C)**, XZ **(E)** and YZ **(F)** planes are shown. **(D)** 3D visualization of automatic image segmentation of the OCM image, with the nucleus marked in red and the nucleolus marked in yellow.

Supplementary Video S5. Mouse prophase I oocyte in transition from the non-surrounded nucleoli (NSN) stage to the surrounded nucleoli (SN) stage. (A) Bright field. **(B)** Hoechst 33342-stained chromatin visualized with fluorescence microscopy. **(C, E, F)** OCM. OCM data obtained with DTIsp #1 resampled to 240x240x240 voxels; consecutive slices in the XY **(C)**, XZ **(E)** and YZ **(F)** planes are shown. **(D)** 3D visualization of automatic image segmentation of the OCM image, with the nucleus marked in red and the nucleolus marked in yellow.

Supplementary Video S6. Mouse prophase I oocyte at the surrounded nucleoli (SN) stage. (A) Bright field. **(B)** Hoechst 33342-stained chromatin visualized with fluorescence microscopy. **(C, E, F)** OCM. OCM data obtained with DTIsp #1 resampled to 240x240x240 voxels; consecutive slices in the XY **(C)**, XZ **(E)** and YZ **(F)** planes are shown. **(D)** 3D visualization of automatic image segmentation of the OCM image, with the nucleus marked in red and the nucleolus marked in yellow.

Supplementary Video S7. Mouse metaphase II oocyte with a spindle placed perpendicularly to the XY plane. (A) Bright field. **(B)** Hoechst 33342-stained chromatin visualized with fluorescence microscopy. **(C, E, F)** OCM. OCM data obtained with DTIsp #3 resampled to 240x240x240 voxels; consecutive slices in the XY **(C)**, XZ **(E)** and YZ **(F)** planes are shown. **(D)** 3D visualization of automatic image segmentation of the OCM image, with the spindle marked in red.

Supplementary Video S8. Mouse metaphase II oocyte with a spindle placed parallel to the XY plane. (A) Bright field. **(B)** Hoechst 33342-stained chromatin visualized with fluorescence microscopy. **(C, E, F)** OCM. OCM data obtained with

DTIsp #3 resampled to 240x240x240 voxels; consecutive slices in the XY **(C)**, XZ **(E)** and YZ **(F)** planes are shown. **(D)** 3D visualization of automatic image segmentation of the OCM image, with the spindle marked in red.

Supplementary Video S9. Nocodazole-treated metaphase II oocyte that lacks a metaphase spindle. (A) Bright field. **(B)** Hoechst 33342-stained chromatin visualized with fluorescence microscopy. **(C, E, F)** OCM. OCM data obtained with DTIsp #3 resampled to 240x240x240 voxels; consecutive slices in the XY **(C)**, XZ **(E)** and YZ **(F)** planes are shown. **(D)** 3D visualization of automatic image segmentation of the OCM image, with the remains of metaphase spindle marked in red.

Supplementary Video S10. Formation and movement of pronuclei in a mouse zygote subjected to time-lapse imaging. (A) Bright field. **(B-D, F-K)** OCM data obtained with DTIsp #4 (300 V-sets acquired every 30 s for approximately 2.5 hrs). **(B)** XY, **(C)** XZ and **(D)** YZ slices from the OCM data processed with Procedure #1. Selected slices processed with Procedure #2 follow the position of the male and female pronuclei in the XY **(F, G)**, XZ **(H, I)** and YZ **(J, K)** planes. All images were resampled to 240x240 pixels. **(E)** 3D visualization of automatic image segmentation of the OCM image obtained with Procedure #1, with the female pronucleus marked in pink, the male pronucleus marked in blue, and the nucleoli marked in yellow.

Supplementary Video S11. 3D visualization of pronuclear trajectories in a mouse zygote tracked using our custom algorithm. The same OCM dataset as in Supplementary Video 10 was used for this analysis. The female pronucleus and its trajectory are marked in pink, the male pronucleus and its trajectory are marked in blue, and the nucleoli are marked in yellow.

Supplementary Video S12. Formation and movement of pronuclei in a nocodazole-treated mouse zygote subjected to time-lapse. (A) Bright field. **(B-D, F-K)** OCM data obtained with DTIsp #4 (300 V-sets acquired every 30 s for approximately 2.5 hrs). **(B)** XY, **(C)** XZ and **(D)** YZ slices from OCM data processed with Procedure #1. Selected slices follow the position of the male pronuclei in the **(F, G)** XY, **(H, I)** XZ and **(J, K)** YZ slices from the OCM data processed with Procedure #2 follow the position of the male and female pronuclei in the XY **(F, G)**, XZ **(H, I)** and YZ **(J, K)** planes. All of the images were resampled to 240x240 pixels. **(E)** 3D visualization of automatic image segmentation of the OCM image obtained with Procedure #1, with the female pronucleus marked in pink, the male pronucleus marked in blue, and the nucleoli marked in yellow. Nocodazole treatment leads to the accumulation of membranous structures in the zygote, which are visible in the OCM as white dots.

Supplementary Video S13. 3D visualization of pronuclear trajectories in a nocodazole-treated mouse zygote tracked using our custom algorithm. The same OCM dataset as in Supplementary Video 12 was used for this analysis. Nocodazole inhibits movement of the pronuclei towards each other and the cell center. The female pronucleus and its trajectory are marked in pink, the male pronucleus and its trajectory are marked in blue, and the nucleoli are marked in yellow.

Supplementary Video S14. Mouse embryo at 1- to 2-cell transition subjected to a time-lapse imaging. (A) Bright field. **(B-D, F-H)** OCM data obtained with DTIsp #5 (400 V-sets acquired every 120 s for 13.5 hrs). **(B)** XY, **(C)** XZ and **(D)** YZ slices from the OCM data processed with Procedure #1. XY **(F)**, XZ **(G)** and YZ **(H)** slices from the OCM data processed with Procedure #2. All images were resampled to 240x240 pixels. **(E)** 3D visualization of automatic image segmentation of the OCM image obtained with Procedure #1, with the female pronucleus marked in dark blue, the male pronucleus marked in red, the spindle marked in cyan, the nuclei of the 2-cell embryo marked in light blue, and the nucleoli marked in yellow.

Supplementary Tables

Supplementary Table S1. Differential time interval scanning protocols (DTIsp) used in our studies. See Supplementary Figure S1 for an explanation of the symbols.

	V-scan					V-set		4D V-set		Comb. 4D V-set		
**DTIsp name**	**T_{Ascan} [us]**	**N_{Ascan}**	**T_{Bscan} [ms]**	**N_{Bscans}/ N_{cBscan}**	**$N_{cBscans}$**	**T_{Vscan} [s]**	**N_{Vscan}**	**T_{Vset} [s]**	**N_{Vset}**	**N_{4DVset}**	**Vset size [GB]**	**Total size [GB]**
DTIsp #1	20	500	11	2	500	-	1	22	10	10	1.9	19
DTIsp #2	20	240	5.8	1	240	1.5	10	30	10	10	2.2	22
DTIsp #3	20	300	6.4	2	70	1	10	30	10	10	1.6	16
DTIsp #4	20	300	6.4	2	70	1	10	30	300	10	1.6	480
DTIsp #5	20	300	6.4	2	70	1	10	120	400	10	1.6	640

Supplementary Table S2. Averaging techniques used with the differential time interval scanning protocols (DTIsp).

	Temporal averaging			Spatial averaging	
**Procedure name**	**V-scan**	**V-set**	**Comb. 4D V-set**	**Slice width [µm]**	**Algorithm**
Procedure #1	Mean	Mean	Mean	-	-
Procedure #2	Mean	Mean	Mean	15	minimum intensity projection
Procedure #3	Mean	Mean	Mean	70	maximum intensity projection

Supplementary Figures

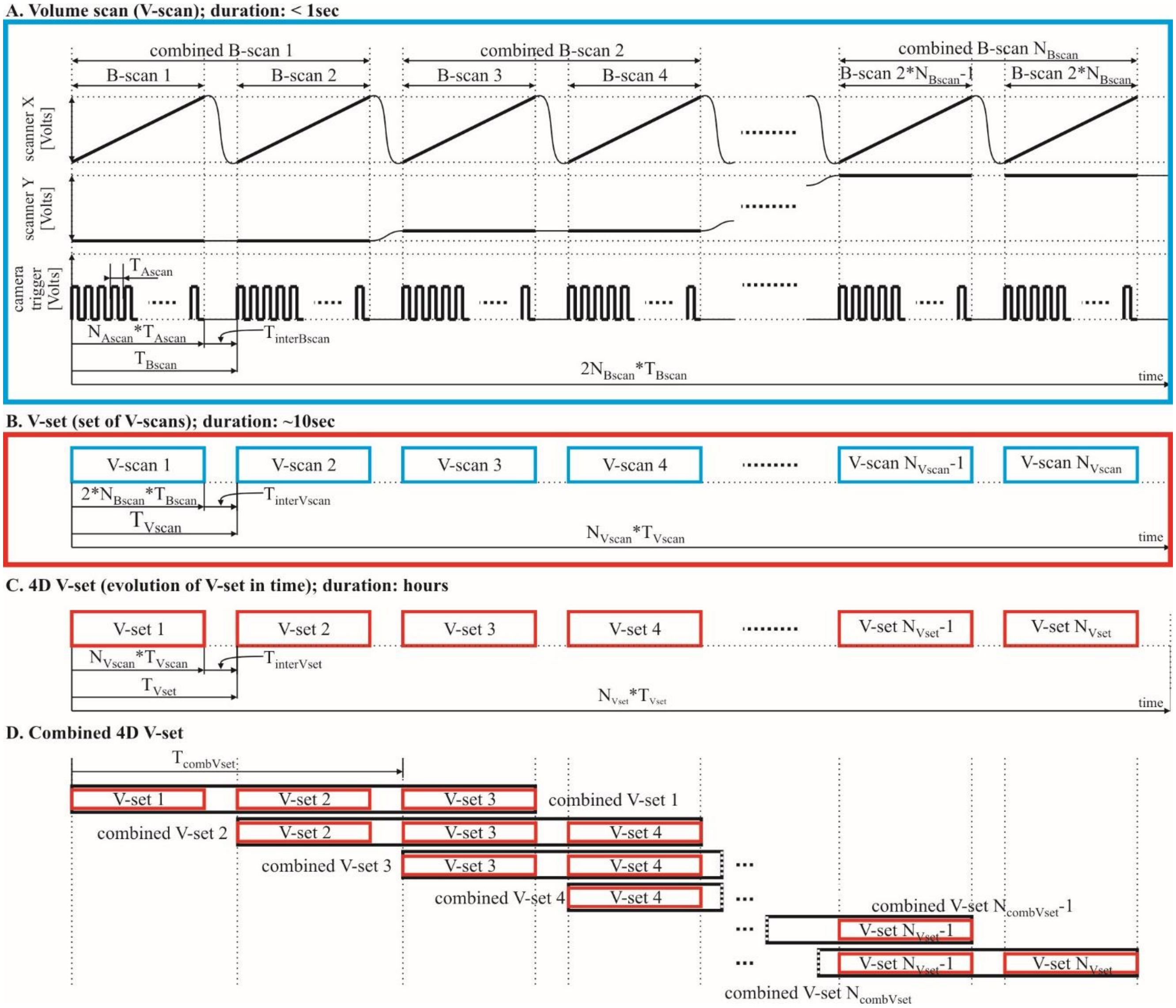

Supplementary Figure S1. Scanning protocols for OCM. (A) Electrical signals used to synchronize a pair of galvanometric scanners that deflected the OCM light beam (scanner X & scanner Y) and the light spectrum acquisition events (camera trigger). One A-scan (line with information about light scattering encoded in amplitude) is generated from each acquisition event. Consecutive acquisitions of A-scans are conducted during constant motion of the light beam along the X lateral direction to create a B-scan (a tomogram with brightness encoding information about the quantity of scattered light). Two B-scans are acquired at the same lateral Y position and are used to create one combined B-scan. This technique is repeated for all lateral Y positions, leading to a 3D distribution of the scattered light V-scan (volume tomogram). **(B)** A set of such volumes is acquired in timespans of approximately one second. This set is used to form one 3D tomogram (V-set) with increased image contrast due to speckle averaging compared with the single V-scan. **(C)** V-sets are acquired over prolonged periods of time (tens of hours) to observe the 3D dynamics of cells inside the V-scan (4D V-set). **(D)** The V-sets forming the 4D V-set are processed using the sliding window technique to further reduce the speckle noise. A new averaged 3D image (a combined V-set) is generated from several V-sets. The next combined V-set is created from the same number of V-sets, but is shifted in time (usually by one V-set). This procedure reduces the time resolution and ability to track fast processes but critically improves the image quality.

Supplementary Information

Diversified Time Interval Scanning Protocol (DTIsp)

For the purposes of this study, we introduced a diversified time interval scanning protocol (DTIsp) that supports multiple time intervals between the acquisitions of consecutive OCM measurements (see Supplementary Figure S1). DTIsp enables researchers to perform a complex analysis of biological processes with different dynamics that occur in oocytes and embryos at the expense of data oversampling. Nonetheless, the oversampled data can be averaged to reduce speckles and produce high-quality structural images or were transformed with a wide spectrum of mathematic operations. The latter included operations such as difference, mean value, maximal value, minimal value or standard deviation and allowed us to attenuate or enhance particular structures on final OCM images.

We designed DTIsp protocols to support up to four different time intervals at which each single point within the 3D volume could be analyzed. Shortest time step, given by the CMOS camera repetition rate, defined the rate at which single axial scans (A-scans) are collected (T_{Ascan}). For all of our measurements T_{Ascan} was set to 20 us. To create a 2D cross-section image (B-scan) A-scans were consecutively acquired while beam was scanning the sample along the X lateral direction. Period at which we collected B-scans (T_{Bscan}) depended on number of A-scans and user defined inter B-scan time offset. At least two B-scans were acquired at the same Y position to be merged into a single combined B-scan. The procedure was repeated for all lateral Y positions and V-scans (3D volume scan) were acquired with T_{Vscan} period. A set of V-scans measured in timespan of approximately one second were averaged into one 3D tomogram (V-set) with increased contrast due to speckle reduction compared to single non-averaged V-scan. To study 3D cell dynamics V-sets were consecutively acquired at T_{Vset} (>20s) period during prolonged measurements (tens of hours). To further reduce the speckle noise, we formed 4D V-sets using sliding window technique. A combined V-sets were generated from several V-sets shifted in time (usually by one T_{Vset}). The latter approach, when applied, improves the image quality. However, time resolution and the ability to track fast cell processes is highly reduced in this case.

Automated Tracking of Zygote Dynamics

The cell segmentation procedure consists of several phases (see Supplementary Figure S2) aligned with the hierarchical structure of the cell: 1) extraction of the cell body, 2) detection of pronuclei, spindle or nuclei (depending on the phase of the process), and 3) segmentation of nucleoli. Each of these procedures requires experimental tuning of multiple parameters on the available sequences of 3D volumes produced by OCM. Once tuned, the procedures are fully automatic and capable of adapting to the varying characteristics of the frames and only require the intervention of an expert in the initial stage, where s/he is asked to roughly select the locations of both the male and female pronuclei in a single image (frame) in the sequence of 3D frames. Beginning with this frame, the algorithm traces the above-mentioned cellular structures forward and backward in time. For clarity, the description that follows refers to consecutive frames as 'previous', 'current', and 'next'; however, in practice, this order will be reversed when the structures are tracked backward in time (in which case, the procedures operate in the same way, except for monitoring the fusion of the pronuclei into the spindle and then the division to two separate nuclei).

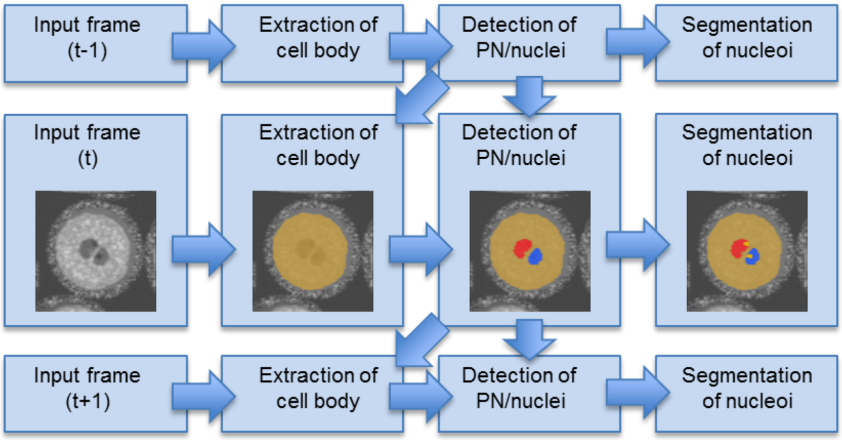

Supplementary Figure S2. Workflow of the cell segmentation algorithm. The segmented cell body is marked in orange, the pronuclei are marked in red and blue, and the nucleoli are marked in yellow.

In the preprocessing stage, we replaced the raw voxel values with their logarithms to compensate for the high dynamic range of the OCT signal.

1) We first blur the 3D image using a Gaussian filter with $\sigma = 0.8$ voxels to segment the cell body. Next, for each 2D layer at a given depth z (for each z coordinate) in the 3D frame, we calculate a separate threshold, tz, using Otsu's method[1]. To make the segmentation more robust, tz is then averaged with the analogous thresholds obtained for the 50 nearest layers, i.e., from $tz-25$ to $tz+25$, resulting in the adjusted threshold tz'. Next, the thresholds are used to classify the voxels in each layer; the voxels in the zth layer with values greater than tz' are assumed to represent the cell. Because some cell structures, like pronuclei, are much darker than the cell body in OCM, their voxels may have lower values than tz' and form 3D 'holes' (cavities) in the thresholding outcome. We label all such holes as cell bodies to obtain a continuous 3D region of adjacent labeled voxels that includes these structures. We then apply a morphological opening to remove the very small or narrow parts of the cell body. This process may lead to several isolated objects, the largest of which (in terms of the volume) is assumed to be the cell body.

When the male pronucleus is very close to the cell border, the adjacent cell membrane may not be clearly rendered, and the above procedure may lead to an apparent cavity on the cell surface. We incorporate the information on the locations of pronuclei in the previous frame (estimated using the method described below in 2), when available, to overcome this problem. First, we identify the points located on the junction of three regions: the cell body detected in the current frame, the pronuclei detected in the previous frame, and the background detected in the current frame. Next, we build a 3D convex hull on these junction voxels, and label all points in the hull as belonging to the cell body. Finally, we fill with labels any cavities that might have resulted from that process; thus, the result is guaranteed to form a continuous 3D region of adjacent labeled voxels.

2) Once the cell body is extracted from a frame, we detect the pronuclei or a spindle, depending on the phase of cell cycle being analyzed. In both cases, the procedure works almost identically; therefore, we refer to both pronuclei and a spindle as 'objects' in the subsequent steps. The detection algorithm only considers the voxels labeled as the cell body by the cell segmentation procedure presented in (1). For each object detected in the previous frame, we calculate the Otsu threshold ot from the corresponding voxels in the current frame. The threshold is slightly decreased to $ot' = 0.85 \cdot ot + 0.15 \cdot mbv$, where mbv is the mean value of voxels below ot (background voxels).

For the frames that contain the spindle, the distribution of voxel values in the cell body is usually significantly different from the distribution in the remaining frames, which required us to cap the threshold at a fixed value of 9.2. Additionally, because the voxels near the cell surface are a bit darker than those throughout the cell interior, we slightly increased their values such that they are less likely to be labeled as a pronucleus.

For each object identified in the previous frame, we approximate it with a small ellipsoid and scale it down. The voxels in the current frame inside the ellipsoid then form 'seeds' for the watershed segmentation algorithm described below. First, we insert all seed points, the information about the object's label (e.g., 'male pronuclei') and the lowest possible priority into a priority queue. Next, we obtain the head element, i.e., the point with the lowest priority and its required label from the queue. If the extracted point is not a seed and fewer than three of its neighbors either have the same label or are candidates to receive the same label, then this point is only marked as a candidate to receive that label and is removed from the queue. In the other case, the point is definitively labeled, and all of its neighbors are added to the priority queue. The priority of each added point is calculated as the absolute difference between the value of the just labeled point and that of the added point. We ignore all points with values above the ot' threshold and points that have been already labeled. The process is repeated until the priority queue is empty.

Once watershed segmentation is completed, we apply the morphological closing operation to the labeled voxels, fill any 3D cavities in the labeled objects (in the same way as when segmenting the cell body in 1), and finally keep only the objects that contain a center of any object detected in the previous frame. Each object segmented in this way is then approximated with an ellipsoid; among others, that ellipsoid is used to seed the watershed segmentation in the next frame, as mentioned above.

Next, we verify whether the center of each ellipsoid lies inside the object detected in the previous image. If not, the corresponding object is labeled as a non-pronucleus (implying that it may represent other cell structures).

As mandated by the underlying biological process, the pronuclei merge at a certain time point to form the spindle, which requires special handling. We merge two pronuclei and label it as a spindle if (i) they have adjacent voxels and (ii) there is no clear partition between them in terms of voxel values. We temporarily merge both adjacent pronuclei into one region and apply the Multi Otsu method with two thresholds to verify the latter condition[2]. Next, we threshold the merged region with

the lower of the resulting Otsu thresholds. If the thresholding leads to one continuous region, it is labeled as a spindle; otherwise, the pronuclei are still considered independent.

We monitor the changes in the shape of each traced spindle to detect the potential nuclear division. In this step, we first approximate the positions of the spindle in the previous and current frame with ellipsoids, and retrieve their longest axes, L_{prev} and L_{curr}, respectively. If $L_{curr} + 5 < L_{prev}$ and $L_{curr} / L_{curr} < 0.7$, we consider these values as a significant change in the shape and/or volume of a spindle, and attempt to detect the objects again. Therefore, we apply a procedure based on the Multi Otsu method[2] and watershed segmentation described above. If the procedure succeeds at detecting two new objects that overlap with one spindle in the previous frame, nuclear division is detected.

3) The last step of automatic image segmentation is to detect the nucleoli inside the pronuclei or nuclei (in case of 2-cell embryos). This procedure processes each pronucleus or nucleus (i.e., object) separately. First, we calculate the average voxel value m of the given object, and set all voxels outside the object to m. Next, adaptive thresholding is used to detect the darker areas, which are considered potential nucleoli. The threshold is calculated by convolving the image with a Gaussian filter and subtracting a constant. Finally, we apply the morphological closing operation to merge the regions that are close to each other. Each resulting isolated region is considered a separate nucleolus.

When carefully tuned, these methods successfully detected all required cellular structures in the sequences of raw OCT frames. Only one sequence required additional manual marking of areas that should not be classified as part of the pronucleus. However, only one frame in that sequence had to be marked in this way; the markers were then properly propagated to consecutive frames and thus no other frames required manual marking.

References

1 Otsu, N. Threshold selection method from gray-level histograms. *IEEE Trans. Syst. Man. Cyb.* **9**, 62-66 (1979).

2 Liao, P.S., Chew, T.S. & Chung, P.C.A fast algorithm for multilevel thresholding. *J. Inf. Sci. Eng.* **17**, 713-727 (2001).
